# MyoPath: A deep learning pipeline for objective morphometric assessment of skeletal muscle biopsies

**DOI:** 10.1016/j.jpi.2026.100747

**Published:** 2026-10-06

**Authors:** Huahua Zhong, Mingshi Gao, Shang Ma, Wenhan Zhang, Nachuan Cheng, Kexin Jiao, Bochen Zhu, Jie Song, Chong Yan, Dongyue Yue, Jianying Xi, Zunguo Du, Wenhua Zhu, Chongbo Zhao, Sushan Luo

**Affiliations:** a Huashan Rare Disease Centre, Department of Neurology, Huashan Hospital, Fudan University, Shanghai, China; b Shanghai Key Laboratory of Gene Editing and Cell Therapy for Rare Diseases, Fudan University, Shanghai, China; c Department of Pathology, Huashan Hospital, Fudan University, Shanghai, China; d Department of Neurology, Renji Hospital, Shanghai Jiaotong University School of Medicine, Shanghai, China

**Keywords:** Skeletal muscle pathology, Digital pathology, Morphometry, Deep learning, Biomarker, Neuromuscular disease

## Abstract

**Aims:**

Muscle biopsy assessment relies on descriptive features that are difficult to compare across samples. We developed MyoPath to quantify these features and examined their usefulness in clinical biopsies and population-based autopsy material.

**Methods:**

MyoPath combines Cellpose-SAM and conventional image analysis within QuPath to segment myofibers, fat, nuclei, and connective tissue. We analyzed 478 hematoxylin and eosin images from HuashanMuscle (79 snap-frozen biopsies) and GTEx (399 formalin-fixed, paraffin-embedded autopsy samples). Thirty-seven features were summarized by seven pathology indicators. A composite model trained on GTEx healthy and wasting strata was transferred to HuashanMuscle without retraining.

**Results:**

Segmentation Dice coefficients ranged from 0.87 to 0.95 on annotated cryosections. Nuclear centralization index (NCI) and fiber size coefficient of variation (fiber CV) discriminated myopathy from controls in HuashanMuscle (AUC 0.85 and 0.79) and wasting from healthy donors in GTEx (0.74 and 0.74). Their GTEx associations changed little after age and sex adjustment. The composite achieved AUC 0.788 (95% CI 0.71–0.85) in GTEx and 0.873 (0.78–0.94) in HuashanMuscle. In the latter cohort, mean predicted probability was 0.49 compared with an observed myopathy proportion of 0.79.

**Conclusions:**

NCI and fiber CV provide interpretable measures of muscle pathology in two distinct tissue settings. MyoPath supports quantitative comparison between groups; clinical severity grading and interpretation of absolute scores require further validation.

## Highlights

• MyoPath quantifies four tissue compartments in H&E images using Cellpose-SAM without task-specific training.
• Seven pathology indicators summarize 37 features; NCI and fibre CV consistently distinguish the two cohorts.
• A GTEx-trained composite transferred to HuashanMuscle without retraining (AUC 0.873, 95% CI 0.78–0.94).
• Absolute scores require calibration and validation against independent clinical severity measures.

## Introduction

Skeletal muscle biopsy is a standard part of the diagnostic workup for patients with neuromuscular symptoms^1^, ^2^, ^3^ and is increasingly used to monitor treatment response in clinical trials and to characterize muscle health in ageing and systemic disease. Interpreting these biopsies requires integrating histomorphological findings with clinical presentation, and the diagnostic yield depends on the specific clinical question.^2^ Pathological evaluation of hematoxylin and eosin (H&E)-stained sections relies on qualitative descriptors such as “mild fiber size variation,” “occasional centralized nuclei,” and “moderate fatty replacement” that resist standardization across observers and institutions.^1^, ^2^, ^3^ Whereas pattern-based approaches to muscle biopsy interpretation have been well described,^2^ no internationally accepted quantitative grading system exists for H&E-based skeletal muscle pathology, in contrast to established grading systems in surgical pathology such as the Gleason system for prostatic adenocarcinoma.^4^

Quantitative measurements are needed for comparisons between patients and for evaluating treatment response. Histopathological endpoints are already used in muscular dystrophy studies,^5^, ^6^ whereas quantitative magnetic resonance imaging (MRI) can track muscle involvement.^7^, ^8^ In DM1, MRI fat fraction correlates with CTG repeat length and clinical severity scales.^8^ Histology provides complementary information at the fiber level, including nuclear position and variation in fiber size.

Computational pathology automates measurements such as Ki-67, PD-L1, and mitotic counts in oncology.^9^ Tools for skeletal muscle morphometry include SMASH,^10^ MyoVision,^11^ MuscleJ,^12^ and Open-CSAM.^13^ Deep learning has extended these approaches to fiber classification^14^ and whole-section analysis with synthetic training data.^15^ Nuclear centralization is a familiar feature of several muscle diseases,^16^ and fiber size variation is routinely assessed in muscle biopsy interpretation.^2^ Quantifying these features alongside tissue composition may make H&E sections more useful for comparisons across clinical and research settings.^3^, ^17^

MyoPath extends fiber analysis to four tissue compartments within a single H&E section. We applied it to two cohorts with different tissue preparation and clinical context: HuashanMuscle hereditary myopathies and biopsy-normal controls, and the GTEx postmortem health-status strata. We asked which of seven interpretable indicators discriminated groups in both settings, and whether a model developed in GTEx retained discrimination when applied to HuashanMuscle. Clinical correlations and additional GTEx analyses were used to examine the scope and limitations of these measurements.

## Materials and methods

### Study design

This retrospective, cross-sectional morphometric study analyzed H&E-stained skeletal muscle sections from two independent cohorts. The HuashanMuscle cohort was collected at Huashan Hospital, Fudan University, under institutional review board approval [IRB number 2022-913]. The GTEx cohort was accessed through the Genotype-Tissue Expression Project (dbGaP accession phs000424). All procedures conformed to the Declaration of Helsinki.

### HuashanMuscle clinical cohort

The cohort comprised 79 skeletal muscle biopsies from patients with genetically confirmed myopathies and controls with normal histopathology enrolled at Huashan Hospital, Fudan University, Shanghai, China (Table 1). Disease groups included DM1 (*n* = 28; 15 female; mean age 42.4 ± 10.7 years),^18^ LGMDR1 (calpainopathy, CAPN3 mutations; *n* = 12; 4 female; mean age 38.6 ± 13.3 years),^19^ LGMDR2 (dysferlinopathy, DYSF mutations; *n* = 22; 8 female; mean age 32.0 ± 11.6 years),^20^ and controls (*n* = 17; 10 female; mean age 41.2 ± 15.0 years). Controls underwent diagnostic biopsy for suspected neuromuscular disease but had no histopathological abnormalities; they were not healthy volunteers. LGMD nomenclature follows the reformed ENMC classification.^21^ Clinical data included CTG repeat count (DM1, *n* = 20), grip strength and 10-m walk time (DM1, *n* = 12 each), and mutation classification for LGMD.

**Table 1:** Study cohort characteristics.

Cohort	Group	n	M/F	Age range	Muscle site	Processing
HuashanMuscle	Control	17	7/10	16–67	Biceps/vastus lat.	Snap-frozen
HuashanMuscle	DM1	28	13/15	26–65	Biceps/vastus lat.	Snap-frozen
HuashanMuscle	LGMDR1	12	8/4	19–66	Biceps/vastus lat.	Snap-frozen
HuashanMuscle	LGMDR2	22	14/8	15–60	Biceps/vastus lat.	Snap-frozen
GTEx	Healthy	107	85/22	20–79	Gastrocnemius	FFPE (postmortem)
GTEx	General Dis.	223	143/80	20–79	Gastrocnemius	FFPE (postmortem)
GTEx	Wasting Dis.	69	48/21	20–79	Gastrocnemius	FFPE (postmortem)

HuashanMuscle controls had diagnostic biopsies without histopathological abnormalities; myopathy groups had established molecular diagnoses. GTEx samples were collected at autopsy. Of 1001 GTEx images, 399 were suitable for transverse muscle analysis. One general-disease sample lacked a computable fiber CV, leaving 398 complete cases (107 healthy, 222 general diseases, and 69 wasting diseases).

### GTEx population cohort

The GTEx project provided skeletal muscle H&E whole-slide images from 1001 postmortem donors.^22^ Of these, 399 had transverse-oriented muscle suitable for analysis; 602 were excluded for longitudinal or tangential sectioning, or insufficient tissue quality. We retained the analysis dataset's health-status strata, within the framework of our previous study.^23^ Healthy donors (*n* = 107) had fast deaths without an atrophy flag; general-disease donors (*n* = 223) were ventilator cases without an atrophy flag; wasting donors (*n* = 69) had intermediate/slow deaths or an atrophy flag. These are proxy groups, not clinical muscle diagnoses. The cohort included 276 males and 123 females aged 20–79 years. One general-disease sample lacked a computable fiber CV, leaving 398 complete cases (107/222/69). Forty samples had a recorded pathology category; 32 included atrophy. These annotations were examined descriptively, without treating them as independent validation of the grouping labels.

### Whole-slide image acquisition

For the HuashanMuscle cohort, open biopsies were obtained from biceps brachii or vastus lateralis muscle. Specimens were snap-frozen in isopentane cooled by liquid nitrogen following standard procedures.^3^ Serial cryosections were cut at 8 μm thickness using a Leica CM 1850 cryostat. Sections were stained with H&E according to routine protocols and digitized using an Olympus VS200 whole-slide scanner at ×40 magnification.

GTEx muscle was collected at autopsy, formalin-fixed and paraffin-embedded, with H&E sections prepared at participating biobanks.^22^ The Hardy scale describes circumstances of death rather than the postmortem interval itself. Nuclear distortion, fragmentation, and retraction spaces were observed in the autopsy sections. Because the connective compartment is obtained by subtraction, non-tissue spaces within an ROI can contribute to its estimated area.

One 1500 × 1500 μm ROI was placed per slide by a single operator in a transversely sectioned area, avoiding folds, tears, staining artifacts, and specimen edges. Morphometric output was not consulted during placement. Selection was not formally blinded to cohort or diagnosis.

### Automated four-tissue segmentation pipeline

A four-tissue pipeline was implemented in QuPath^24^ with Cellpose-SAM integration^25^, ^26^ (Fig. 1). Deep learning was used for myofiber instance segmentation; conventional image analysis was used for fat and nuclei, followed by calculation of the residual connective compartment. Processing a 1500 × 1500 μm ROI took approximately 5 min on a single GPU.

**Fig. 1:**
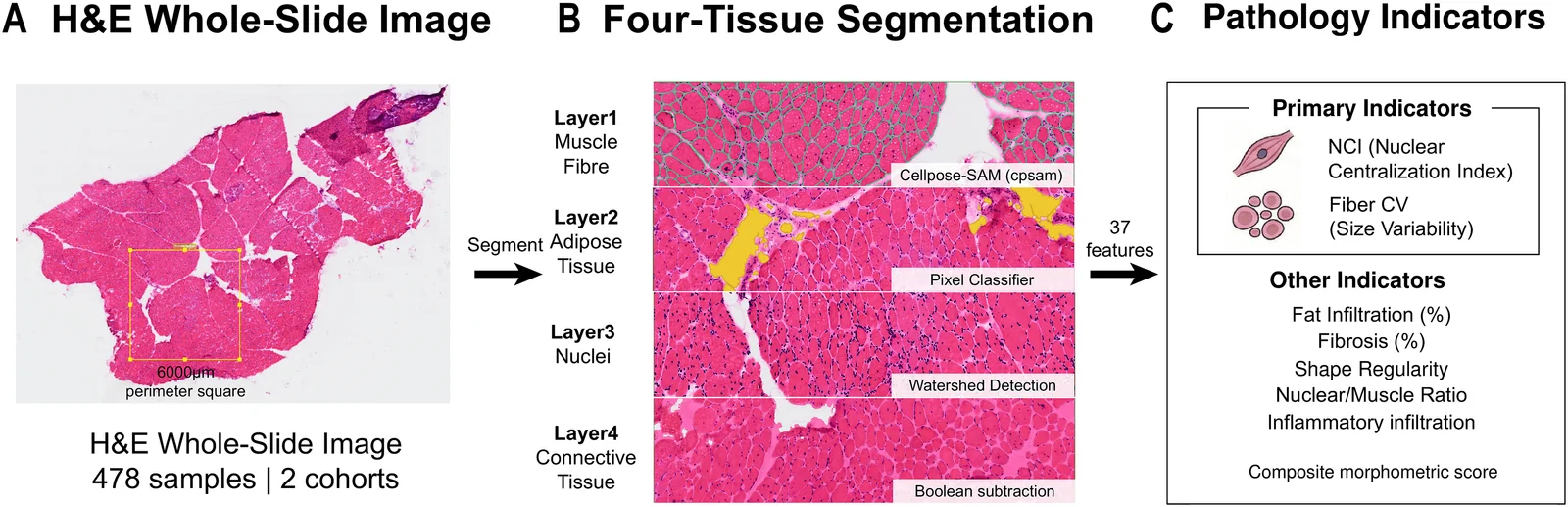
MyoPath workflow. (A) H&E image with a 1500 × 1500 μm ROI (6000-μm perimeter); 478 images from two cohorts were processed. (B) Myofibers are segmented with Cellpose-SAM, fat with a pixel classifier and nuclei with watershed detection. The connective compartment is obtained by subtraction. (C) Seven pathology indicators summarize 37 extracted features. NCI and fiber CV are highlighted for their consistent cross-cohort discrimination.

*Layer 1 (myo*fiber *instances):* Myofibers were segmented with the pretrained Cellpose-SAM model (cpsam),^26^ which incorporates the Segment Anything Model backbone.^27^ The weights were not fine-tuned. Processing settings were fixed before analysis: expected fiber diameter 100 μm, 4× downsampling, and 500-pixel tile overlap, with GPU acceleration. These settings were not selected using the validation annotations or the discriminative results reported here.

*Layer 2 (fat infiltration):* Adipose tissue was identified using a pretrained QuPath pixel classifier distinguishing fat from myofibers and connective tissue by H&E staining intensity. The minimum area threshold was 50 μm^2^.

*Layer 3 (nuclei):* Nuclei were detected with QuPath WatershedCellDetection on the hematoxylin channel after colour deconvolution (area range 10–500 μm^2^, intensity threshold 0.4, Gaussian sigma 1.5 μm).

*Layer 4 (connective tissue):* Connective area was calculated as ROI area minus myofiber area minus effective fat area. Nuclear area was not subtracted separately, because nuclei lie within these tissue compartments. The residual includes endomysial and perimysial tissue and serves as a proxy for fibrosis. Under-segmentation of muscle or fat, or non-tissue space within the ROI, can increase this estimate; there is no separate artifact mask.

Myofibers, fat, and the residual connective compartment define tissue areas; the nuclear layer provides an overlapping cellular measurement. Their annotations can be analyzed separately or together.

### Morphometric feature extraction

Thirty-seven morphometric features were extracted per sample, comprising tissue areas, fiber size statistics, nuclear counts and positions, and derived pathology indicators. Full definitions and formulas are provided in Supplementary File 1.

Seven indicators were specified a priori by their correspondence to features assessed during muscle biopsy evaluation. The subsequent screen of all 37 features described their redundancy and discriminative performance; it did not determine membership of the panel:

1. *NCI:* The mean relative radial position of nuclei within myofibers, reflecting nuclear centralization.^3^, ^16^, ^28^ Nuclear centroids were assigned to the fiber polygon containing them, or to the residual connective compartment if located there; the remainder were unassigned. NCI averages boundary distance divided by equivalent fiber radius, capped at 1, over nuclei successfully localized within fibers. Supplementary File 1 gives the formula and denominator.
2. *Fiber size variability coefficient (*fiber *CV):* The standard deviation divided by the mean of myofiber cross-sectional areas, describing variation in fiber size.^3^, ^10^, ^29^
3. *Fiber shape regularity:* Mean circularity (4πA/P^2^; 1 = perfect circle), with lower values indicating less regular fiber outlines.^30^
4. *Fat infiltration (%):* Fatty tissue as a percentage of ROI area. A late-stage marker of dystrophic or denervation injury.^31^, ^32^
5. *Fibrosis (%):* Connective tissue as a percentage of ROI area, reflecting endomysial and perimysial fibrotic remodeling.^33^
6. *Nuclear/muscle ratio:* The number of nuclei within myofibers divided by fiber count. An increase may accompany regeneration or other changes in cellularity; H&E alone cannot distinguish the contributing cell processes.^34^
7. *Inflammatory infiltration:* The pipeline label for connective-tissue nuclear density (nuclei/mm^2^). It measures interstitial cellularity without identifying the cell types present.^35^, ^36^

The panel covers nuclear position, fiber size and shape, tissue replacement, and cellularity. NCI was preferred to a binary central-nucleus ratio because it retains continuous positional information; fiber CV is dimensionless. The later designation of NCI and fiber CV as primary indicators was descriptive, based on their cross-cohort performance. All seven indicators entered the composite model.

### Segmentation quality assessment

Two pathologists (M.G. and S.M.) independently annotated myofibers, fat, and nuclei on 30 randomly selected ROIs from 10 HuashanMuscle samples (5 myopathy, 5 control). Disagreements were resolved by consensus. Pixel-level Dice and intersection-over-union (IoU) were calculated against the consensus reference for each tissue compartment. Agreement of morphometric measurements was assessed by two-way random-effects, absolute-agreement ICC. Within-sample variation was summarized across five non-overlapping ROIs for NCI, fiber CV, fat, and fibrosis. The reference annotations were confined to cryosections and were not used to tune the pipeline; the 10 samples remained in the group comparisons.

### Statistical analysis

Group comparisons used Mann–Whitney or Kruskal–Wallis tests, with Bonferroni correction for pairwise comparisons. Effect sizes were rank-biserial correlations. Clinical associations used Spearman correlation; the 21 DM1 comparisons are reported as exploratory, with unadjusted *p*-values and Benjamini–Hochberg FDR results.^37^ Ordered GTEx trends used the Jonckheere–Terpstra test. AUC intervals used 2000 outcome-stratified bootstrap resamples; within-cohort AUC differences used paired resamples. Logistic models assessed age- and sex-adjusted associations and the information added by the seven indicators beyond demographics (likelihood-ratio test). GTEx age brackets were represented by midpoints; exact ages were used for HuashanMuscle. Hardy categories had insufficient overlap across the GTEx groups for reliable adjustment. General-versus-healthy differences were therefore described separately, using age- and sex-adjusted linear models. FDR correction was applied across the eight annotation-related comparisons.

A logistic model combining the seven indicators was trained on GTEx healthy versus wasting donors (*n* = 176) and applied to HuashanMuscle controls versus myopathies (*n* = 79) without retraining. The MyoPath Score is defined as:

$$\text{MyoPath Score}=1/\left(1+e^{-z}\right)$$

z ≈ −9.17 + 27.11 × NCI + 3.96 × fiber CV + 8.22 × shape regularity +0.017 × fat infiltration (%) + 0.043 × fibrosis (%) + 0.123 × nuclear/muscle ratio + 1.31 × 10^−6^ × inflammatory infiltration (nuclei/mm^2^).

The model used L2-regularized logistic regression (C = 1.0) on features standardized with GTEx training-set means and standard deviations. The displayed raw-scale coefficients are rounded; full-precision parameters are provided in Supplementary Data 1. Standardized coefficients express contributions per standard deviation. Leave-one-out cross-validation assessed internal discrimination. Coefficient stability was described by 1000 stratified bootstrap refits, with percentile intervals and sign consistency; multicollinearity was assessed by VIF. Calibration was assessed by the Brier score and a logistic recalibration model estimating intercept and slope jointly^38^; observed proportions were plotted against mean predicted probabilities. HuashanMuscle was the external transfer cohort; the GTEx general-versus-healthy comparison was internal. Analyses used Python (scipy, scikit-learn) and R, with two-sided tests at α = 0.05.

## Results

### Segmentation performance

MyoPath processed all 478 images. In the representative HuashanMuscle ROI (HE_M3807), it delineated 953 myofibers, 52 fat regions, and 1626 nuclei (Fig. 2A). Dice coefficients were 0.92 ± 0.03 for myofibers, 0.95 ± 0.02 for fat, 0.87 ± 0.04 for nuclei, and 0.88 ± 0.04 for connective tissue (Fig. 2B; Table 2). Automated fat and fibrosis measurements correlated with pathologist assessment (rho = 0.989 and 0.946; Fig. 2C). ICCs against the two pathologists were 0.93/0.93 for NCI, 0.93/0.92 for fiber CV, 0.99/0.98 for fat, 0.92/0.89 for fibrosis, and 0.98/0.98 for nuclear/muscle ratio. Shape regularity showed lower agreement (0.27/0.43); connective nuclear density was not included. Mean within-sample CVs across five ROIs were 0.051, 0.054, 0.068, and 0.065 for NCI, fiber CV, fat, and fibrosis, respectively. Single-ROI fat estimates correlated weakly with the pathologists' whole-specimen estimates in 245 GTEx samples (rho = 0.105, 95% CI −0.03–0.23), indicating limited agreement across sampling scales.

**Fig. 2:**
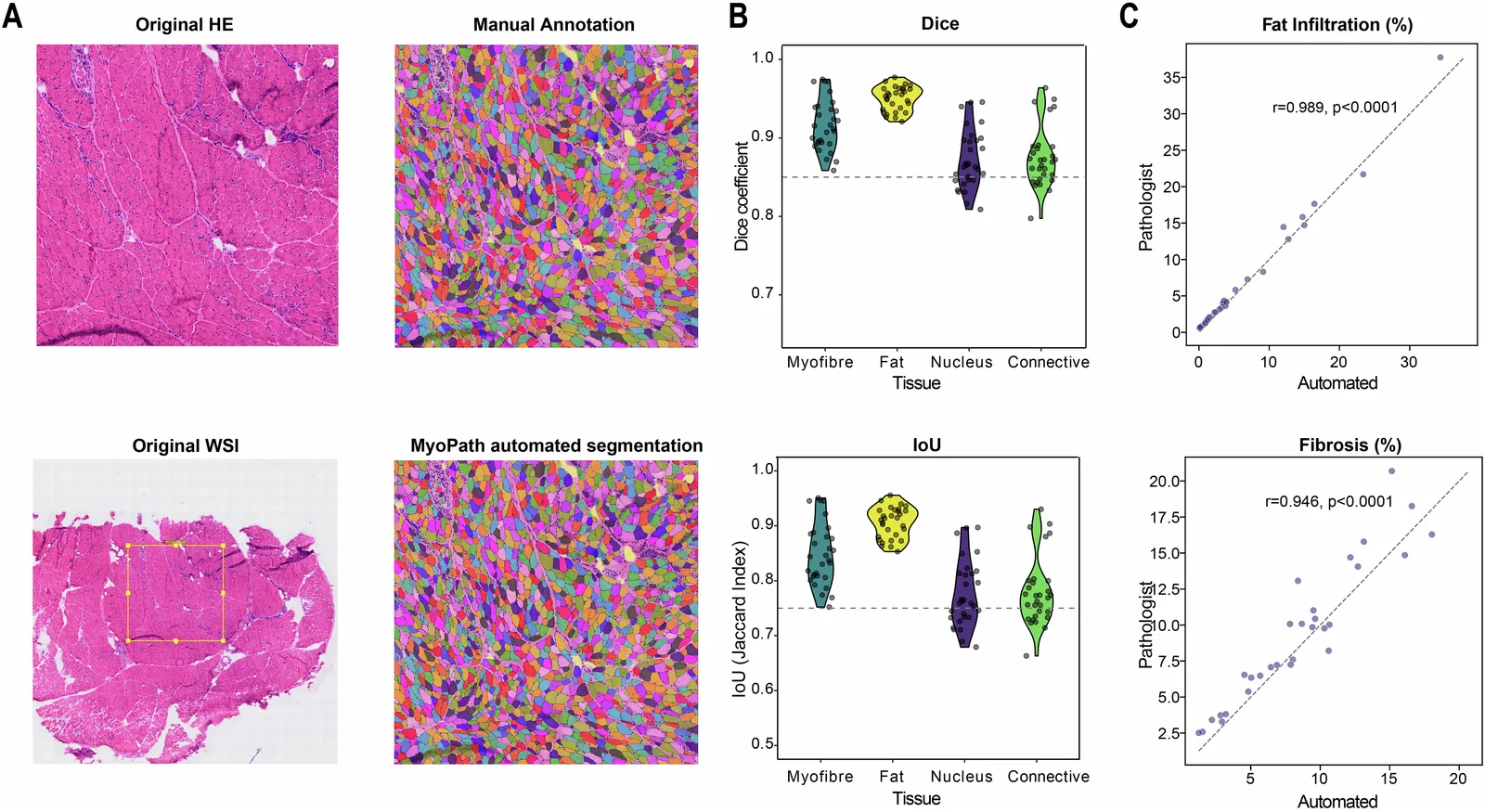
Segmentation validation and observer agreement. (A) Representative sample (HE_M3807). Top row: original H&E image (left) and pathologist manual annotation with each myofiber in a unique color (right). Bottom row: whole-slide image with ROI location (left) and MyoPath automated segmentation (right). (B) Dice coefficients (top) and intersection-over-union (bottom) for four tissue layers (*n* = 30 annotated ROIs). Dashed lines indicate 0.85 (Dice) and 0.75 (IoU) thresholds. (C) Scatter plots comparing automated measurements with pathologist assessment for fat infiltration (Spearman *r* = 0.989, *p* < 0.0001) and fibrosis (*r* = 0.946, *p* < 0.0001). Identity lines shown.

**Table 2:** Segmentation accuracy across four tissue layers.

Tissue layer	Method	Dice coefficient	IoU
Myofiber	Cellpose-SAM	0.92 ± 0.03	0.85 ± 0.06
Fat	Pixel classifier	0.95 ± 0.02	0.91 ± 0.03
Nucleus	Watershed	0.87 ± 0.04	0.78 ± 0.06
Connective	Boolean subtract	0.88 ± 0.04	0.78 ± 0.06

Values are mean ± SD (*n* = 30 annotated ROIs from 10 HuashanMuscle samples). Dice and IoU computed by pixel-level comparison between automated segmentation and consensus annotations from two pathologists.

### Discriminative performance of the 37 features

The pipeline extracted 37 features describing tissue composition, fiber size and shape, and nuclear distribution and location. Each was screened by Mann–Whitney analysis for controls versus myopathies in HuashanMuscle and healthy versus wasting donors in GTEx.

In HuashanMuscle, 15 of 37 features differed between controls and myopathies (unadjusted *p* < 0.05; Fig. 3B). Nuclear-position and cellularity measures ranked highest, including central ratio (AUC 0.91), peripheral ratio (0.88), and total nuclear count (0.88). Fiber CV had AUC 0.79. In GTEx, fiber size and nuclear position were more prominent (Fig. 3C): fiber area Q1 had AUC 0.75, and NCI and fiber CV both had AUC 0.74. Fat and fibrosis each had AUC 0.67. Nuclear/muscle ratio, with AUC 0.83 in HuashanMuscle, had AUC 0.50 in GTEx. The rankings therefore differed between the biopsy and autopsy settings.

**Fig. 3:**
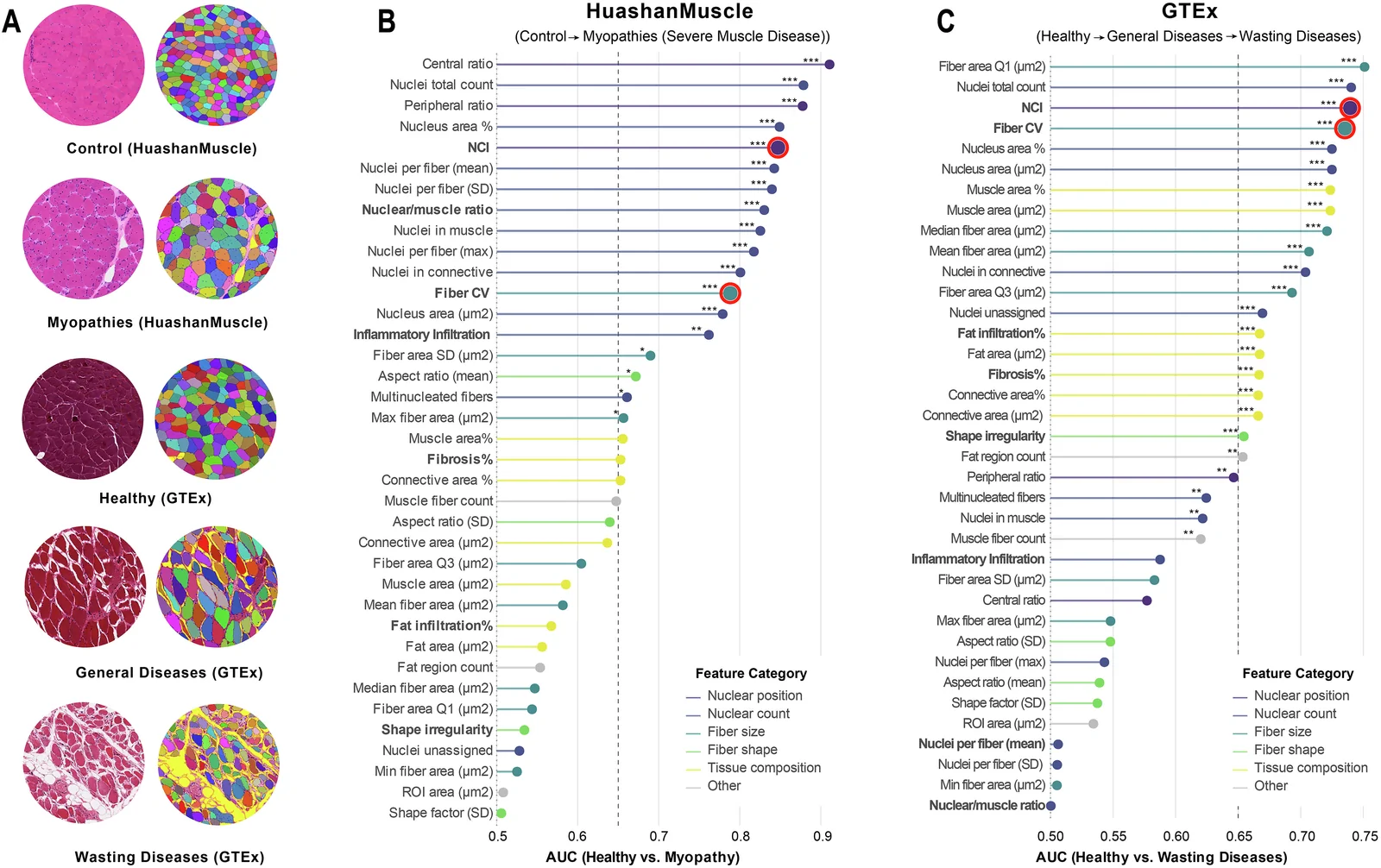
Feature ranking across 37 morphometric features. (A) Representative segmentation results from each diagnostic group. (B) HuashanMuscle cohort: AUC values for discriminating controls from myopathies, ranked by discriminative power. (C) GTEx cohort: AUC values for discriminating healthy donors from those with wasting diseases. Significance: *p < 0.05, **p < 0.01, ***p < 0.001.

These comparisons describe the performance of the prespecified seven-indicator panel. Their differing rankings also show why a feature with a high AUC in one cohort may not be the most useful measure in another.

### Seven pathology indicators across cohorts

Four of the seven indicators differed between HuashanMuscle controls and myopathies (Fig. 4A). NCI was higher in myopathy (median 0.077, IQR 0.062–0.121) than controls (0.049, 0.041–0.058; *p* = 1.3 × 10^−5^, *r* = 0.69), as was fiber CV (0.522 versus 0.369; *p* = 2.9 × 10^−4^, *r* = 0.58). Their AUCs were 0.85 (95% CI 0.75–0.93) and 0.79 (0.67–0.88), respectively (Fig. 8A). Nuclear/muscle ratio and connective nuclear density also differed (*p* = 3.4 × 10^−5^ and 0.003). Fat, fibrosis, and shape regularity did not reach significance (*p* = 0.40, 0.056, and 0.67). The seven indicators added information beyond age and sex (likelihood-ratio *p* = 5.2 × 10^−8^; Supplementary Table S2).

**Fig. 4:**
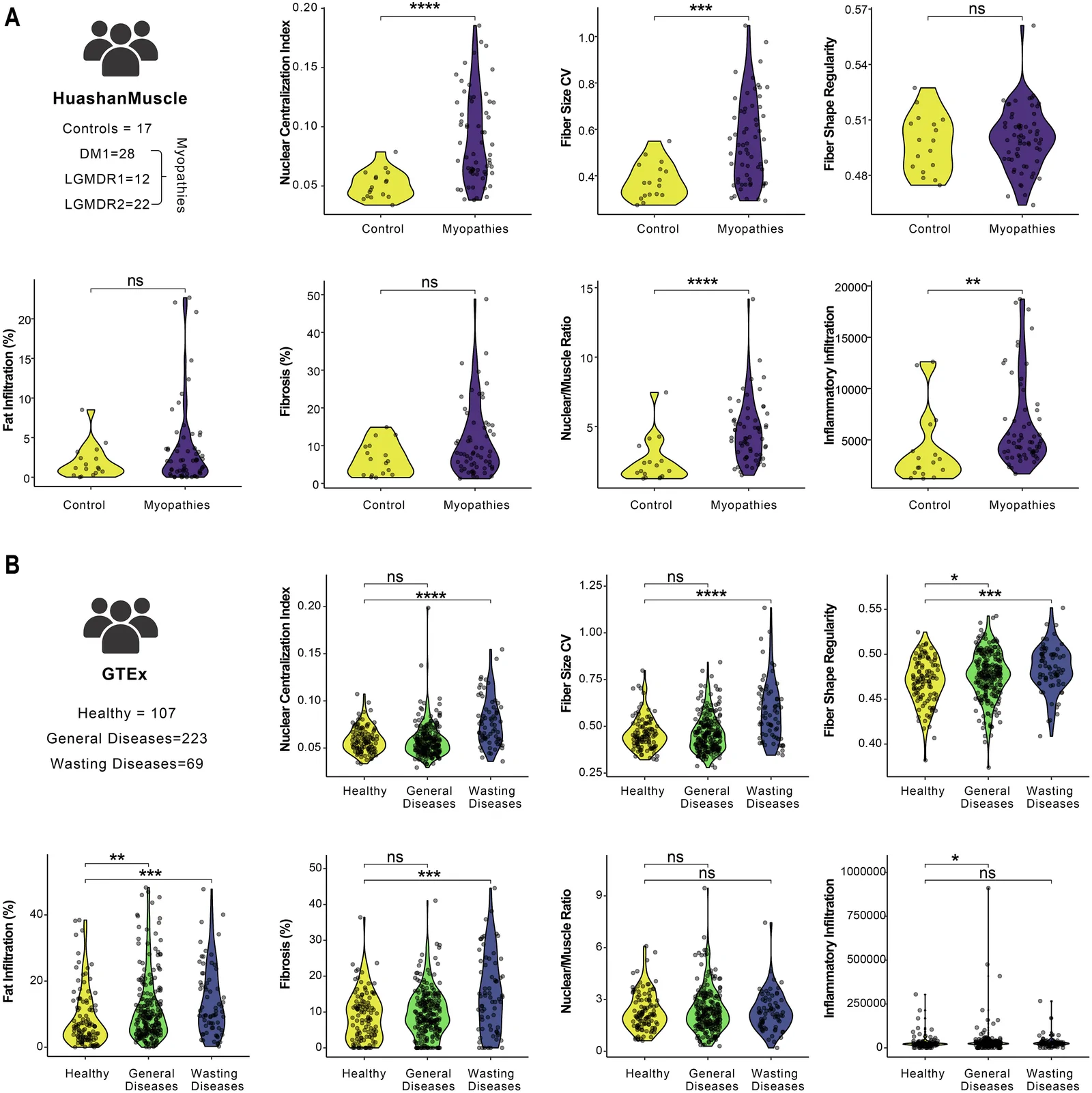
Seven pathology indicators across groups. (A) HuashanMuscle controls (*n* = 17) and myopathies (*n* = 62). (B) GTEx health-status strata. NCI and fiber CV showed ordered trends (Jonckheere–Terpstra *p* = 7.4 × 10^−5^ and 2.0 × 10^−5^), with similar medians in healthy and general-disease donors and higher values in wasting donors.

In GTEx, NCI and fiber CV showed ordered trends across the strata (Jonckheere–Terpstra *p* = 7.4 × 10^−5^ and 2.0 × 10^−5^; Fig. 4B). Medians were similar in healthy and general-disease donors (NCI 0.057/0.054; fiber CV 0.447/0.451) and higher in wasting donors (0.075 and 0.559). Healthy-versus-wasting AUCs were 0.74 for both indicators (95% CI 0.66–0.81 and 0.65–0.81). Age- and sex-adjusted ORs per standard deviation were 3.01 (2.06–4.94) for NCI and 2.96 (2.00–5.16), close to their unadjusted values of 3.08 and 3.05 (Supplementary Table S2). Fat and fibrosis also increased across the strata (*p* < 0.001); nuclear/muscle ratio did not (*p* > 0.05).

NCI and fiber CV were the only two of the seven indicators with AUC above 0.70 in both cohorts. Tissue composition and cellularity measures showed greater variation in discriminative performance between settings.

### Exploratory clinical correlations

We examined clinical associations of NCI and fiber CV in the available DM1 and LGMD subgroups (Fig. 5). These analyses were exploratory because of the small sample sizes.

**Fig. 5:**
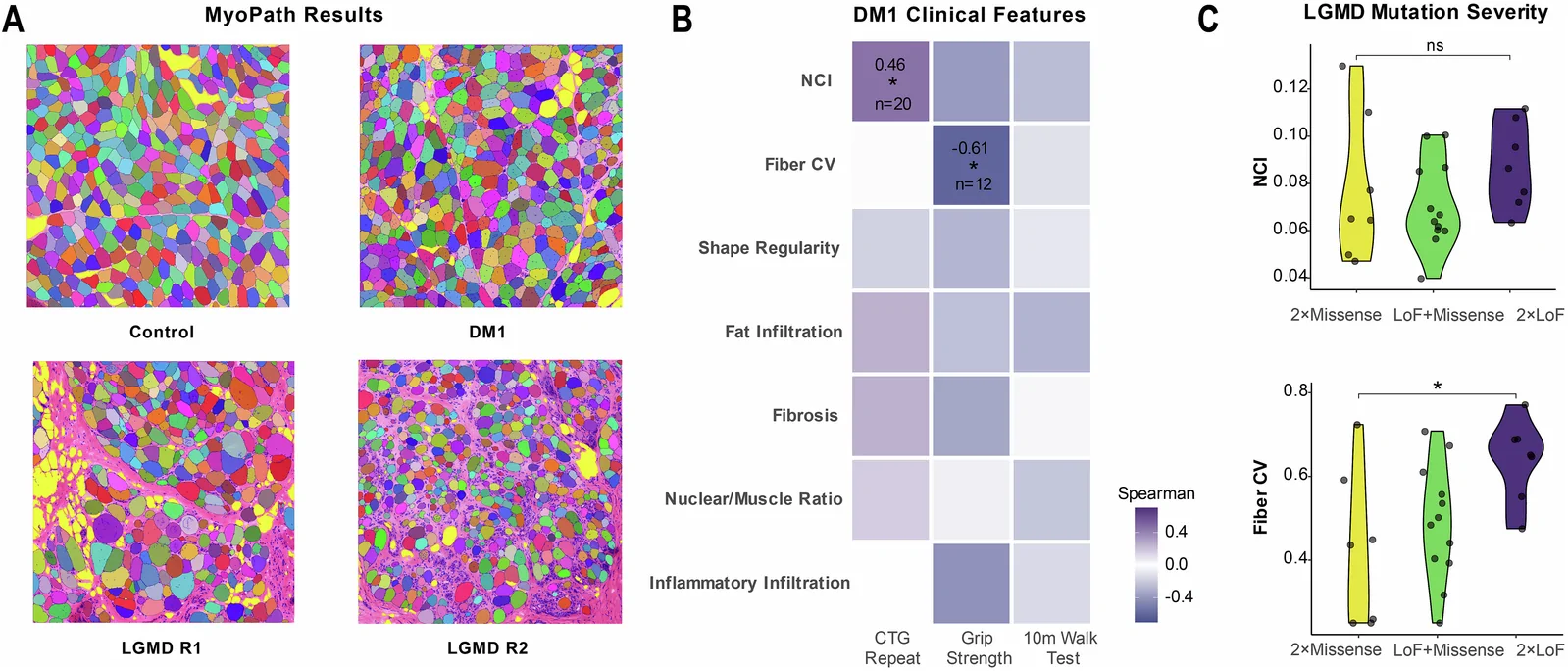
Exploratory clinical and mutation associations. (A) Representative fiber maps. (B) DM1 correlation heatmap; associations are unadjusted and none of the 21 comparisons survived FDR correction. (C) LGMD mutation groups (*n* = 26).

In DM1, NCI correlated with CTG repeat count (rho = 0.46, unadjusted *p* = 0.042, *n* = 20), and fiber CV correlated inversely with grip strength (rho = −0.61, unadjusted *p* = 0.036, *n* = 12; Fig. 5B). None of the 21 indicator–clinical variable correlations remained significant after Benjamini–Hochberg correction (smallest adjusted *p* = 0.44).

Among 26 LGMD patients with confirmed biallelic mutations, median fiber CV was 0.44 for two missense variants, 0.49 for a loss-of-function plus missense variant, and 0.65 for two loss-of-function variants (Kruskal–Wallis *p* = 0.044; Fig. 5C). NCI showed no significant difference (*p* = 0.23). These small-subgroup observations require replication.

### Additional analyses of the GTEx strata

Atrophy was recorded in 32 wasting samples. Within that stratum, the score distinguished flagged from unflagged specimens (AUC 0.76, 95% CI 0.64–0.86), with a similar estimate after exclusion of ventilator cases (0.76, 0.63–0.88; Fig. 7A,C; Supplementary Table S3). These are exploratory comparisons: 35 of 37 unflagged samples had no recorded category, and the annotations do not provide an independent test of the grouping labels.

All healthy donors were fast deaths and all general-disease donors were ventilator cases (Supplementary Table S4). The score showed little discrimination between these two groups (AUC 0.53, 95% CI 0.46–0.60; *p* = 0.31). After age and sex adjustment, fat infiltration was higher in general-disease donors by 0.38 SD (95% CI 0.14–0.62). Corresponding differences were −0.003 SD (−0.24–0.24) for NCI and 0.17 SD (−0.07–0.41) for fiber CV (Fig. 7B). This contrast cannot attribute differences solely to agonal state.

### Comparison of nuclear-position measures

NCI and central ratio describe the same nuclei using continuous and binary positional measures. In GTEx, NCI had the higher AUC (0.74 versus 0.58; paired difference 0.16, 95% CI 0.07–0.26; *p* = 0.0005). In HuashanMuscle, the difference was −0.06 (0.85 versus 0.91; 95% CI −0.15–0.01; *p* = 0.11; Fig. 8B). Several count- and threshold-based nuclear features also differed in performance between cohorts (Supplementary Fig. S1). These comparisons do not isolate the effect of tissue fixation.

### Cross-cohort transfer of the MyoPath score

The seven-indicator composite achieved a training AUC of 0.788 (95% CI 0.71–0.85; LOO-CV AUC 0.735) in 176 GTEx healthy and wasting donors (Fig. 6A). NCI and fiber CV had the largest standardized coefficients (0.57 and 0.53), with bootstrap intervals of 0.11–1.09 and 0.04–1.18. The remaining coefficient intervals included zero. Training-set VIFs were below 2 (Supplementary Table S5).

**Fig. 6:**
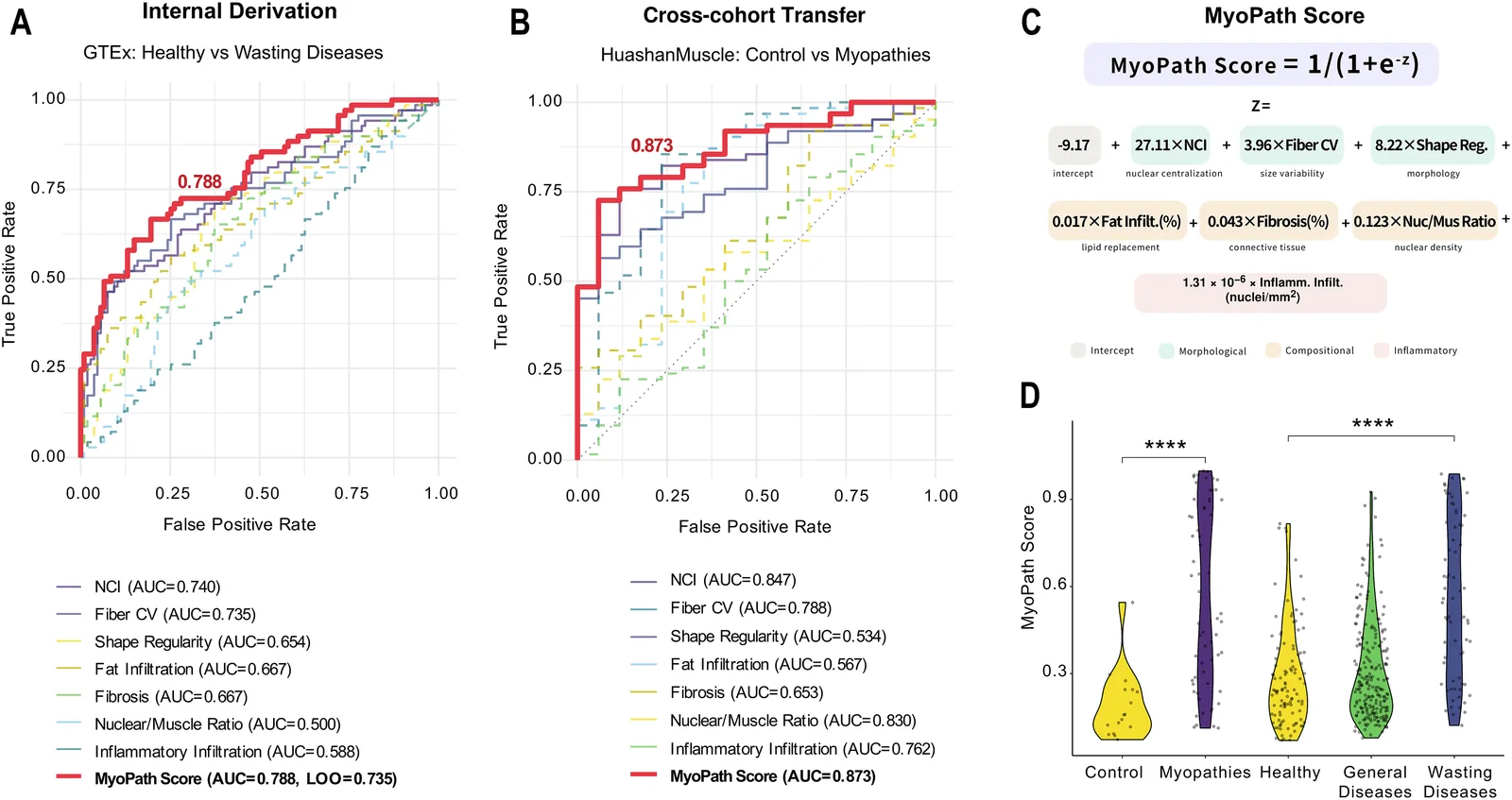
MyoPath Score development and transfer. (A) GTEx training ROC curves: composite AUC 0.788 (95% CI 0.71–0.85), LOO-CV AUC 0.735. (B) Fixed-model transfer to HuashanMuscle: AUC 0.873 (0.78–0.94). (C) Formula with rounded coefficients; Supplementary Data 1 contains full-precision parameters. (D) Score distributions for 477 complete cases. Within-cohort disease-versus-control comparisons used the Mann–Whitney test (both *p* < 0.0001).

**Fig. 7:**
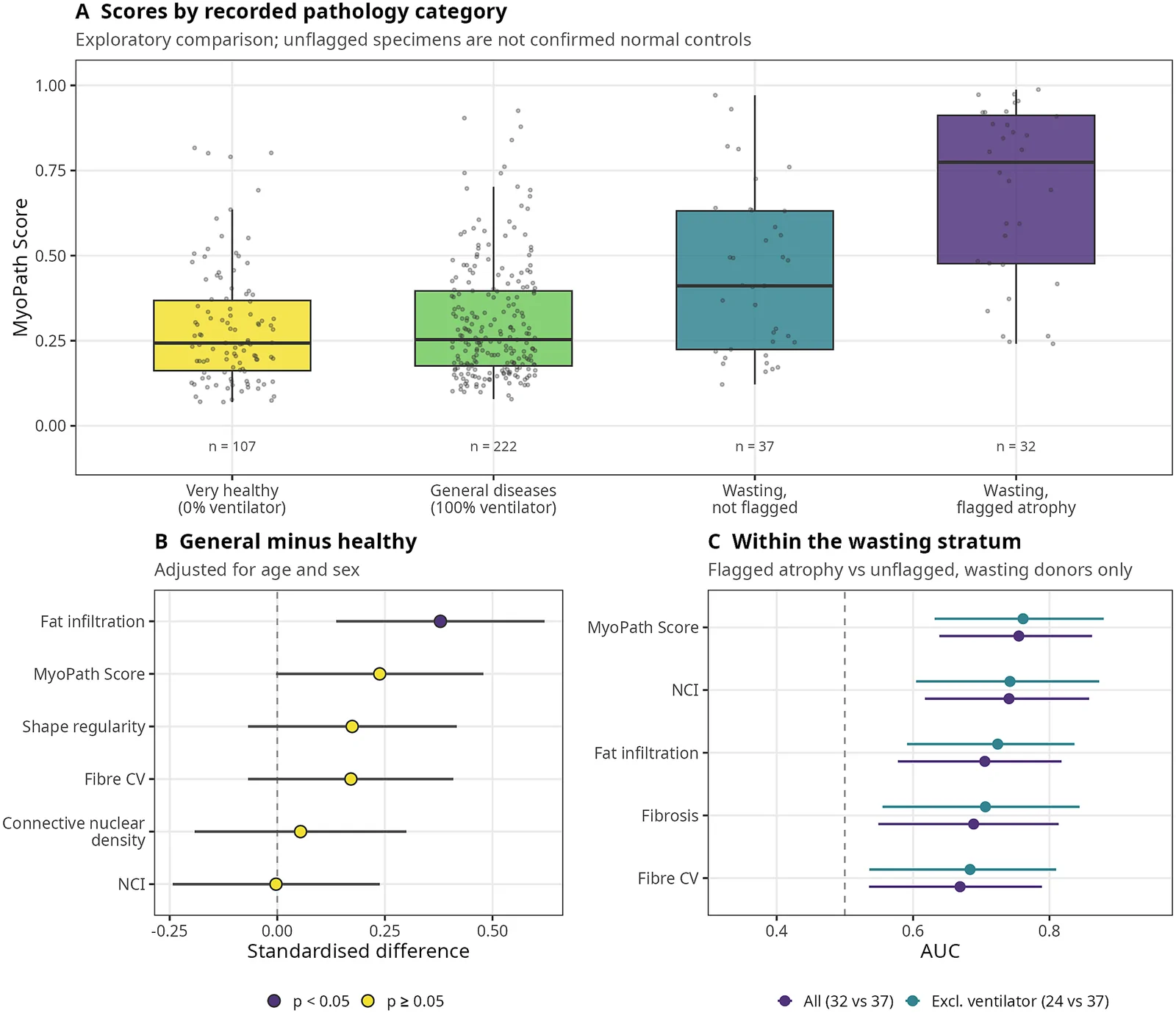
Exploratory comparisons within GTEx. (A) Score distributions, with wasting donors separated by the recorded atrophy category. Boxes show medians and interquartile ranges. (B) General-disease minus healthy differences adjusted for age and sex, with 95% CI; these groups also differ in Hardy category. Symbol fill indicates statistical significance, not evidence of equivalence. (C) AUCs for flagged versus unflagged wasting specimens, including all donors (32/37) and excluding ventilator cases (24/37). Unflagged specimens are not confirmed normal controls; these analyses are not independent validation of the grouping labels.

**Fig. 8:**
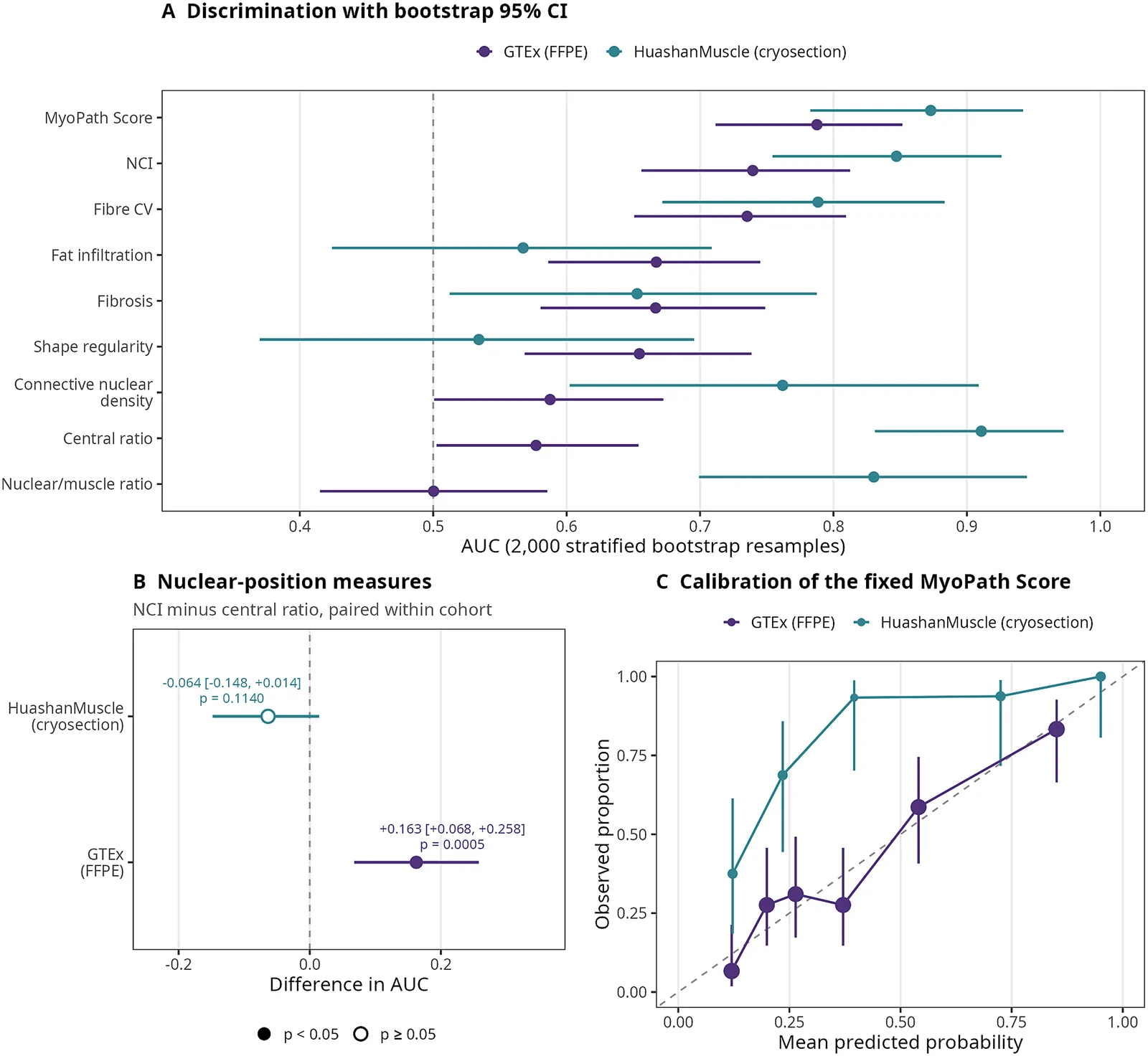
Discrimination, nuclear-position measures and calibration. (A) AUCs with 95% bootstrap CI (2000 stratified resamples). (B) Within-cohort paired differences in AUC between NCI and central ratio. (C) Observed case proportions against mean predicted probability for the fixed model, with Wilson 95% CI. The dashed line indicates perfect calibration.

The fixed model achieved AUC 0.873 (95% CI 0.78–0.94) in HuashanMuscle (Fig. 6B). Its mean predicted probability was 0.49, compared with an observed myopathy proportion of 0.79. Joint logistic recalibration gave an intercept of 2.72 and slope of 1.61; the Brier score was 0.23 (Fig. 8C; Supplementary Table S6). Thus, discrimination transferred across cohorts, whereas the absolute probabilities did not match this biopsy sample.

Among 477 samples with complete indicators, median scores were 0.16 in HuashanMuscle controls, 0.50 in HuashanMuscle myopathies, and 0.24, 0.25, and 0.54 in GTEx healthy, general-disease, and wasting donors (Fig. 6D). Both within-cohort disease-versus-control comparisons were significant (*p* < 0.0001).

## Discussion

MyoPath turns familiar features of muscle biopsy interpretation into measurements that can be compared across samples. NCI and fiber CV showed the most consistent discrimination across clinical cryosections and postmortem FFPE material. The seven-indicator composite retained discrimination when transferred without retraining to a cohort with different diseases, preparation, and controls. These findings support the use of automated H&E morphometry for group comparisons, while leaving clinical severity grading as a separate question.

Fat infiltration and fibrosis are established features of chronic muscle disease,^2^ but their discriminative value differed between cohorts. They also occur with ageing, disuse, and other conditions.^39^ Sampling is an additional consideration: fat and connective tissue are unevenly distributed, and the single-ROI fat measurement agreed only weakly with the GTEx whole-specimen estimate. Retraction spaces and under-segmentation can further increase the residual connective estimate. NCI and fiber CV describe structures within fibers and may be less dependent on the amount of interstitial tissue in a ROI, although their sampling sensitivity still requires study. Whole-muscle imaging, including Dixon fat–water separation,^40^ measures composition at a different scale.

The GTEx findings place these measurements in the broader health-status framework described in our previous study.^23^ The main separation was between wasting donors and the other strata; the healthy and general-disease groups had similar NCI and fiber CV values. The annotation and Hardy analyses provide context for this result, but cannot establish an independent muscle diagnosis or separate the effects of illness, agonal state, and postmortem conditions. The clinical biopsy cohort therefore supplies the more directly defined disease comparison.

Earlier tools, including SMASH, MyoVision, MuscleJ, and Open-CSAM, established automated approaches to muscle fiber measurement.^10^, ^11^, ^12^, ^13^ FiNuTyper^14^ and synthetic-training approaches^15^ extended the use of deep learning in muscle histology. MyoPath adds fat, connective tissue, and nuclear distribution to fiber measurements from the same H&E section. Network-based analysis has been related to pathologist severity scores,^41^ whereas convolutional networks have distinguished inflammatory from hereditary myopathies.^42^ These approaches address complementary tasks: diagnosis, grading, and measurement need different reference standards.

The composite model's transfer AUC supports its use as a summary of morphometric differences. It does not imply that the same score denotes the same clinical severity in both cohorts. The higher HuashanMuscle AUC may reflect its disease and control selection, and its case proportion is not an estimate of population prevalence. Calibration should therefore be reassessed in the intended setting before probabilities are interpreted. NCI and fiber CV had the most stable coefficients, whereas uncertainty in the smaller coefficients limits interpretation of their individual contributions.

Several limitations remain. GTEx health-status labels are proxies, closely linked to death circumstances, and the available pathology annotations do not establish their independence from label assignment. Missing annotations do not indicate normal muscle. The two cohorts also differ in disease, muscle site, and processing, so the nuclear-position comparison cannot isolate fixation effects. Segmentation accuracy was assessed only on cryosections. A single operator selected one ROI per slide without formal blinding to diagnosis or cohort; whole-section or systematically sampled multi-ROI analysis would better address sampling bias. HuashanMuscle was a single-center cohort with biopsy-normal controls, and the clinical correlation and mutation subgroups were small. The DM1 associations did not survive multiple-comparison correction. No independent clinical severity endpoint was used to validate the score.

MyoPath quantifies muscle fibers and their tissue context on routine H&E sections. NCI and fiber CV retained discriminative value in both cohorts, and the fixed composite model transferred to clinical biopsies.

## CRediT authorship contribution statement

H.Z. and M.G. contributed equally to this work. H.Z.: conceptualization, slide preparation, data collection, data analysis, writing—original draft. M.G.: slide preparation, pathological annotation, data collection, writing—original draft. S.M. and W.Z.: data collection, data analysis. N.C., K.J., B.Z., J.S., C.Y., D.Y., J.X., and W.Z.: data collection. C.Z. and S.L.: conceptualization, resources, supervision, funding acquisition. All authors reviewed and edited the manuscript.

## Ethical approval

This study was approved by the Institutional Review Board of Huashan Hospital, Fudan University (IRB number 2022-913). GTEx data were accessed through dbGaP (accession phs000424) under the GTEx project's data access protocols. All procedures conformed to the Declaration of Helsinki. Patient consent to participate was obtained in accordance with institutional requirements.

## Funding

This study was supported by the 10.13039/501100001809National Natural Science Foundation of China (82471426, 82571592), and the Open Research Fund of Shanghai Key Laboratory of Gene Editing and Cell Therapy for Rare Diseases (gect-2025-Z01).

## Declaration of competing interest

The authors declare that they have no known competing financial interests or personal relationships that could have appeared to influence the work reported in this article.

## Data Availability

GTEx data are available through dbGaP (phs000424), with Hardy categories and pathology annotations in the public sample attributes. Supplementary Data 1 provides the full-precision fitted model, de-identified per-sample morphometric features and fixed-model scores for reproducing the main discrimination results. HuashanMuscle images and analysis results are available at https://myotoolkit.huashanmuscle.com. The image-analysis pipeline is available at https://github.com/Hirriririir/MyoPath. Additional data are available from the corresponding authors upon reasonable request.

## Appendix A Supplementary data

Supplementary Fig. S1 Performance of 37 features across cohorts. (A) HuashanMuscle versus GTEx AUCs by feature family. (B) Seven-indicator and composite profiles. (C) Between-cohort AUC differences by measurement type. Cohort differences include disease, tissue preparation and reference definition.

Supplementary material 1 Definitions of the 37 morphometric features. Table S1 lists the seven pathology indicators and 30 underlying descriptive features (tissue composition, fibre size, fibre shape, nuclear distribution and nuclear localisation) with their units, followed by the notation and formulas used to calculate them, including the nuclear centralisation index.

Supplementary material 2 Additional analyses of the seven pathology indicators and the MyoPath Score. Table S2: apparent discrimination of models fitted within each cohort, and GTEx odds ratios per standard deviation. Table S3: exploratory comparisons between atrophy-flagged and unflagged GTEx wasting-disease donors. Table S4: Hardy categories by GTEx stratum, and age- and sex-adjusted differences between general-disease and healthy donors. Table S5: variance inflation factors and bootstrap stability of the standardised model coefficients. Table S6: calibration of the fixed GTEx-trained model in the training set and after transfer to HuashanMuscle.
