# Supplementary material for: MyoPath: A deep learning pipeline for objective morphometric assessment of skeletal muscle biopsies
Source: J Pathol Inform. 2026 Oct 6;23:100747. doi: 10.1016/j.jpi.2026.100747 (PMC13742344; doi:10.1016/j.jpi.2026.100747)
Supplement: Supplementary file 1 [file mmc1.pdf]

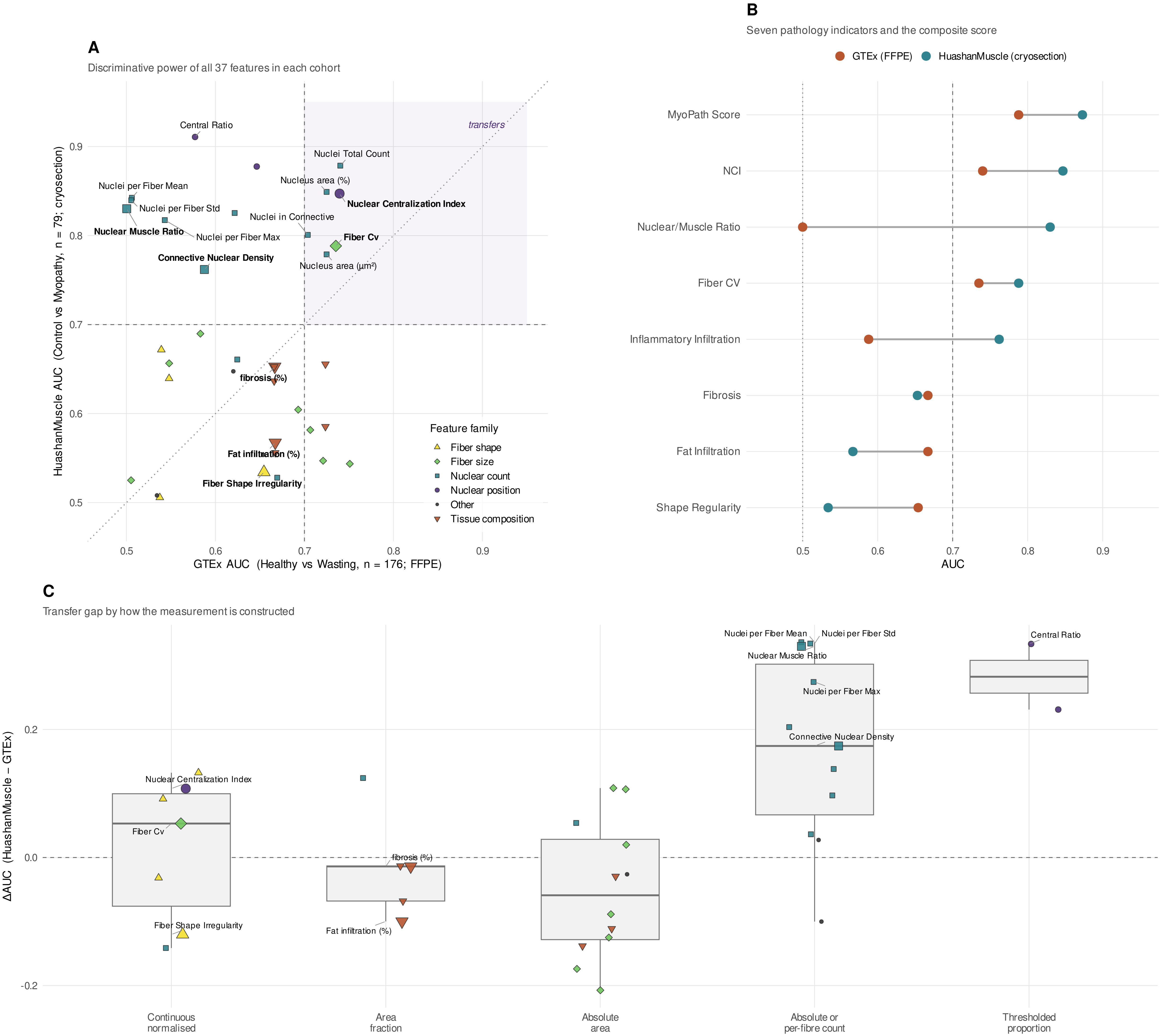

A
Discriminative power of all 37 features in each cohort
transfers
Central Ratio
Nuclei Total Count
Nuclei per Fiber Mean
Nucleus area (%)
Nuclear Centralization Index
Nuclei per Fiber Std
Nuclei in Connective
Nuclear Muscle Ratio
Nuclei per Fiber Max
Fiber Cv
Connective Nuclear Density
Nucleus area (µm²)
fibrosis (%)
Fat infiltration (%)
Fiber Shape Irregularity
Feature family
Fiber shape
Fiber size
Nuclear count
Nuclear position
Other
Tissue composition
HuashanMuscle AUC (Control vs Myopathy, n = 79; cryosection)
GTEx AUC (Healthy vs Wasting, n = 176; FFPE)
B
Seven pathology indicators and the composite score
GTEx (FFPE)
HuashanMuscle (cryosection)
MyoPath Score
NCI
Nuclear/Muscle Ratio
Fiber CV
Inflammatory Infiltration
Fibrosis
Fat Infiltration
Shape Regularity
AUC
C
Transfer gap by how the measurement is constructed
ΔAUC (HuashanMuscle − GTEx)
Continuous normalised
Area fraction
Absolute area
Absolute or per-fibre count
Thresholded proportion
