# Supplementary material for: MyoPath: A deep learning pipeline for objective morphometric assessment of skeletal muscle biopsies
Source: J Pathol Inform. 2026 Oct 6;23:100747. doi: 10.1016/j.jpi.2026.100747 (PMC13742344; doi:10.1016/j.jpi.2026.100747)
Supplement: Supplementary file 2 [file mmc2.docx]

**Supplementary Material**

*MyoPath: A Deep Learning Pipeline for Objective Morphometric Assessment of Skeletal Muscle Biopsies*

This file defines the 37 morphometric features used in the study. Analyses use continuous measurements; no clinical normal ranges or severity cut-offs are established here. Full-precision parameters for the fitted logistic MyoPath Score are supplied in Supplementary Data 1.

## Table S1. Complete Feature List (37 Features)

### 1.1 Seven Pathology Indicators

These 7 features are clinically interpretable indicators corresponding to cardinal features assessed during muscle biopsy evaluation.

| **#** | **Feature** | **Unit** | **What it measures** |
| --- | --- | --- | --- |
| 1 | nuclear_centralization_index | 0–1 | Mean boundary distance divided by equivalent fibre radius, capped at 1, across nuclei successfully localised within fibres. |
| 2 | fiber_cv | dimensionless | Standard deviation divided by the mean of myofibre cross-sectional areas. |
| 3 | fiber_shape_irregularity | 0–1 | Mean circularity of fibre cross-sections (4πA/P²). A circle has value 1; lower values indicate less regular outlines. |
| 4 | fat_infiltration_pct | % | Effective adipose area as a percentage of ROI area, after removing overlap with muscle polygons. |
| 5 | fibrosis_pct | % | Residual area (ROI minus muscle minus effective fat) as a percentage of ROI area. This is a proxy for fibrosis and can include non-tissue spaces. |
| 6 | nuclear_muscle_ratio | dimensionless | Number of nuclei assigned to myofibres divided by fibre count. The measurement does not identify the contributing cell processes. |
| 7 | connective_nuclear_density_per_mm2 | nuclei/mm² | Number of nuclei assigned to the connective compartment per mm² of that compartment. H&E analysis does not establish their cell types. |

### 1.2 Underlying Descriptive Features (30 Features)

### A. Tissue Composition (10 features)

| **#** | **Feature** | **Unit** | **What it measures** |
| --- | --- | --- | --- |
| 8 | roi_area_um2 | um² | Total area of the analyzed region of interest. Serves as the denominator for percentage-based metrics. |
| 9 | muscle_fibers_count | count | Total number of individual myofibers detected by Cellpose-SAM within the ROI. |
| 10 | muscle_area_um2 | um² | Sum of cross-sectional areas of all detected myofibers. |
| 11 | muscle_area_pct | % | Myofibre area as a percentage of ROI area. |
| 12 | fat_regions_count | count | Number of discrete adipose regions identified by the pixel classifier. |
| 13 | fat_area_um2 | um² | Total area occupied by adipose tissue, excluding overlap with muscle fiber annotations. |
| 14 | connective_area_um2 | um² | Area of connective tissue, computed by subtracting muscle and fat from total ROI area. |
| 15 | connective_area_pct | % | Connective tissue as a fraction of ROI area. Equal to fibrosis_pct in the exported study data. |
| 16 | nucleus_area_um2 | um² | Total area occupied by all detected nuclei. |
| 17 | nucleus_area_pct | % | Detected nuclear area as a percentage of ROI area. Nuclei overlap the tissue compartments. |

### B. Fiber Size (7 features)

| **#** | **Feature** | **Unit** | **What it measures** |
| --- | --- | --- | --- |
| 18 | fiber_mean_area_um2 | um² | Mean cross-sectional area of the detected fibres. |
| 19 | fiber_median_um2 | um² | Median cross-sectional area of the detected fibres. |
| 20 | fiber_std_um2 | um² | Standard deviation of fibre areas, using the fibre count as denominator. |
| 21 | fiber_min_um2 | um² | Smallest detected fibre area. |
| 22 | fiber_max_um2 | um² | Largest detected fibre area. |
| 23 | fiber_q1_um2 | um² | 25th percentile of detected fibre areas. |
| 24 | fiber_q3_um2 | um² | 75th percentile of detected fibre areas. |

### C. Fiber Shape (3 features)

| **#** | **Feature** | **Unit** | **What it measures** |
| --- | --- | --- | --- |
| 25 | shape_factor_std | dimensionless | Standard deviation of fibre circularity. |
| 26 | aspect_ratio_mean | dimensionless | Mean ratio of longer to shorter bounding-box side. A value of 1 indicates equal sides. |
| 27 | aspect_ratio_std | dimensionless | Standard deviation of the bounding-box aspect ratios. |

### D. Nuclear Distribution (7 features)

| **#** | **Feature** | **Unit** | **What it measures** |
| --- | --- | --- | --- |
| 28 | nuclei_total_count | count | Total number of nuclei detected by watershed segmentation within the ROI (all tissue compartments combined). |
| 29 | nuclei_in_muscle | count | Nuclei whose centroids fall within myofiber polygons. Includes subsarcolemmal and internal nuclei. |
| 30 | nuclei_in_connective | count | Number of nuclear centroids assigned to the connective compartment. |
| 31 | nuclei_unassigned | count | Nuclei that could not be assigned to muscle or connective tissue. May include nuclei in fat regions or at tissue boundaries. |
| 32 | nuclei_per_fiber_mean | count/fiber | Mean number of nuclei per detected fibre. |
| 33 | nuclei_per_fiber_std | count/fiber | Standard deviation of the number of nuclei per fibre. |
| 34 | nuclei_per_fiber_max | count | Largest number of nuclei assigned to one fibre. |

### E. Nuclear Localization (3 features)

| **#** | **Feature** | **Unit** | **What it measures** |
| --- | --- | --- | --- |
| 35 | peripheral_ratio | dimensionless | Fraction of successfully localised nuclei with relative boundary distance ≤0.3. |
| 36 | central_ratio | dimensionless | Fraction of successfully localised nuclei with relative boundary distance ≥0.7. |
| 37 | multinucleated_fiber_count | count | Number of fibres containing more than one detected nucleus. |

## 2. Notation and Coordinate System

| **Symbol** | **Definition** |
| --- | --- |
| A_ROI | Total area of the region of interest (ROI) in μm² |
| N_f | Total number of detected muscle fibers |
| a_i | Area of the i-th muscle fiber in μm² |
| p_i | Perimeter of the i-th muscle fiber in μm |
| N_nuc | Total number of detected nuclei |
| s | Pixel size (scanner-dependent, default 0.137 μm/pixel) |
| d | Downsample factor applied during segmentation |

All spatial coordinates originate from QuPath image metadata. Pixel-level measurements are converted to physical units:

*A (μm²) = A_pixels × (s × d)²*

*L (μm) = L_pixels × (s × d)*

## 3. Tissue Composition Metrics

### 3.1 Muscle Area

*A_muscle = Σ aᵢ (i = 1 to N_f)*

*muscle_area_pct = (A_muscle / A_ROI) × 100%*

### 3.2 Fat Infiltration

Fat regions are detected by a pre-trained pixel classifier. To avoid double-counting tissue where fat and muscle annotations overlap, the effective fat area is computed as:

*A_fat = Σ area(Fⱼ \ ∪ Mᵢ) [geometric difference]*

*fat_infiltration_pct = (A_fat / A_ROI) × 100%*

where Fⱼ is the j-th fat polygon, Mᵢ is the i-th muscle fiber polygon, and \ denotes the geometric difference operator.

### 3.3 Fibrosis (Connective Tissue)

Connective tissue area is computed by Boolean subtraction of muscle and fat from the total ROI:

*A_connective = A_ROI − A_muscle − A_fat*

*fibrosis_pct = (A_connective / A_ROI) × 100%*

### 3.4 Nucleus Area

*nucleus_area_pct = (Σ aₖⁿᵘᶜ / A_ROI) × 100%*

## 4. Muscle Fiber Size Metrics

### 4.1 Descriptive Statistics

*fiber_mean_area_um2 = ā = (1/N_f) × Σ aᵢ*

fiber_std_um2 = σₐ = √[ (1/N_f) × Σ(aᵢ − ā)² ]

*fiber_median_um2 = median(a₁, a₂, …, a_{N_f})*

*fiber_q1_um2 = Q₁(a), fiber_q3_um2 = Q₃(a)*

*fiber_min_um2 = min(aᵢ), fiber_max_um2 = max(aᵢ)*

### 4.2 Fiber Size Variability (Coefficient of Variation)

*fiber_cv = fiber_size_variability_cv = σₐ / ā*

### 4.3 Equivalent Diameter

*dᵢᵉᑫ = √(4aᵢ / π)*

## 5. Muscle Fiber Shape Metrics

### 5.1 Shape Factor (Circularity)

*shape_factor_i = 4π aᵢ / pᵢ²*

*shape_factor_mean = fiber_shape_irregularity = (1/N_f) × Σ shape_factor_i*

A perfect circle has shape factor 1.0; lower values indicate less regular outlines.

### 5.2 Aspect Ratio (Elongation)

For the bounding box of each fiber polygon with width wᵢ and height hᵢ:

*aspect_ratio_i = max(wᵢ, hᵢ) / min(wᵢ, hᵢ)*

*aspect_ratio_mean = (1/N_f) × Σ aspect_ratio_i*

A value of 1.0 indicates equal bounding-box width and height; larger values indicate elongation.

## 6. Nuclear Distribution Metrics

### 6.1 Nucleus Assignment

Each detected nucleus centroid cₖ is classified by spatial containment testing (Shapely contains):

*location(k) = muscle	if ∃ i: Mᵢ ∋ cₖ*

*connective	if cₖ ∈ (A_ROI \ ∪Mᵢ \ ∪Fⱼ)*

*unassigned	otherwise*

*N_nuc^muscle = #{k : location(k) = muscle}*

*N_nuc^conn = #{k : location(k) = connective}*

### 6.2 Nuclei per Fiber

For each fiber Mᵢ, the number of contained nuclei:

*nᵢ = #{k : Mᵢ ∋ cₖ}*

*nuclei_per_fiber_mean = (1/N_f) × Σ nᵢ*

*multinucleated_fiber_count = #{i : nᵢ > 1}*

### 6.3 Nuclear/Muscle Ratio

*nuclear_muscle_ratio = N_nuc^muscle / N_f*

### 6.4 Connective Tissue Nuclear Density

*connective_nuclear_density_per_mm² = N_nuc^conn / (A_connective / 10⁶)*

where A_connective is in μm² and 10⁶ converts to mm².

## 7. Nuclear Centralization Index (NCI)

NCI summarises the position of nuclei localised within myofibres.

### 7.1 Relative Radial Position

For each nucleus k within fiber Mᵢ:

*dₖᵇᵒᵘⁿᵈᵃʳʸ = min_{p ∈ ∂Mᵢ} ‖cₖ − p‖*

*rᵢᵉᑫ = √(aᵢ / π)*

*ρₖ = min( dₖᵇᵒᵘⁿᵈᵃʳʸ / rᵢᵉᑫ, 1.0 )*

where dₖᵇᵒᵘⁿᵈᵃʳʸ is the Euclidean distance from the nucleus centroid to the nearest point on the fiber boundary, rᵢᵉᑫ is the equivalent circle radius, and ρₖ ∈ [0, 1] (ρₖ = 0 at the boundary; larger values indicate more inward positions).

### 7.2 Aggregate NCI

*NCI = nuclear_centralization_index = (1/N_analyzed) × Σ ρₖ*

where N_analyzed is the number of nuclei successfully localized within muscle fibers.

### 7.3 Peripheral and Central Ratios

Nuclei are classified into three zones based on their relative radial position:

*zone(k) = peripheral	if ρₖ ≤ 0.3*

*intermediate	if 0.3 < ρₖ < 0.7*

*central	if ρₖ ≥ 0.7*

*peripheral_ratio = #{k : ρₖ ≤ 0.3} / N_analyzed*

*central_ratio	= #{k : ρₖ ≥ 0.7} / N_analyzed*
