# Supplementary material for: MyoPath: A deep learning pipeline for objective morphometric assessment of skeletal muscle biopsies
Source: J Pathol Inform. 2026 Oct 6;23:100747. doi: 10.1016/j.jpi.2026.100747 (PMC13742344; doi:10.1016/j.jpi.2026.100747)
Supplement: Supplementary file 3 [file mmc3.docx]

**MyoPath — Supplementary Tables S2–S6**

**S2a. Apparent discrimination of models fitted within each cohort.**

| **Cohort** | **Model** | **n** | **Cases** | **AUC (95% CI)** |
| --- | --- | --- | --- | --- |
| GTEx | Age + sex | 176 | 69 | 0.611 (0.522–0.692) |
| GTEx | Seven indicators | 176 | 69 | 0.787 (0.712–0.851) |
| GTEx | Indicators + age + sex | 176 | 69 | 0.789 (0.714–0.852) |
| HuashanMuscle | Age + sex | 79 | 62 | 0.586 (0.444–0.721) |
| HuashanMuscle | Seven indicators | 79 | 62 | 0.947 (0.892–0.990) |
| HuashanMuscle | Indicators + age + sex | 79 | 62 | 0.956 (0.903–0.993) |

AUC intervals: 2,000 stratified bootstrap resamples. Likelihood-ratio tests for indicators added to age and sex: GTEx χ²=48.1, df=7, p=3.5×10⁻⁸; HuashanMuscle χ²=47.2, df=7, p=5.2×10⁻⁸. These within-cohort refits are distinct from transfer of the fixed GTEx model (HuashanMuscle AUC 0.873).

**S2b. GTEx odds ratios per standard deviation (n=176).**

| **Indicator** | **Unadjusted OR (95% CI)** | **Age/sex-adjusted OR (95% CI)** |
| --- | --- | --- |
| NCI | 3.08 (2.12–4.94) | 3.01 (2.06–4.94) |
| Fibre CV | 3.05 (2.06–5.20) | 2.96 (2.00–5.16) |
| Shape regularity | 1.78 (1.26–2.66) | 1.80 (1.26–2.73) |
| Fat infiltration | 1.72 (1.26–2.56) | 1.65 (1.20–2.50) |
| Fibrosis | 2.08 (1.50–3.09) | 2.22 (1.58–3.52) |
| Nuclear/muscle ratio | 0.98 (0.71–1.32) | 0.96 (0.69–1.31) |
| Connective nuclear density | 1.05 (0.73–1.70) | 1.01 (0.70–1.61) |

Unpenalised logistic regression; intervals from 1,000 bootstrap refits. HuashanMuscle ORs are not shown because estimates were unstable with 17 controls.

**S3. Exploratory comparisons within GTEx wasting donors.**

| **Indicator** | **All: AUC (95% CI)** | **FDR p** | **No ventilator: AUC (95% CI)** | **FDR p** |
| --- | --- | --- | --- | --- |
| MyoPath Score | 0.76 (0.64–0.86) | 0.0023 | 0.76 (0.63–0.88) | 0.005 |
| NCI | 0.74 (0.62–0.86) | 0.0025 | 0.74 (0.60–0.87) | 0.0062 |
| Fat infiltration | 0.71 (0.58–0.82) | 0.0094 | 0.72 (0.59–0.84) | 0.009 |
| Fibrosis | 0.69 (0.55–0.81) | 0.0145 | 0.71 (0.56–0.84) | 0.0141 |
| Fibre CV | 0.67 (0.54–0.79) | 0.0262 | 0.68 (0.54–0.81) | 0.0263 |
| Shape regularity | 0.63 (0.49–0.76) | 0.0887 | 0.62 (0.46–0.77) | 0.164 |
| Nuclear/muscle ratio | 0.50 (0.35–0.64) | 0.976 | 0.51 (0.37–0.66) | 0.865 |
| Connective nuclear density | 0.49 (0.35–0.63) | 0.968 | 0.52 (0.37–0.67) | 0.865 |

All donors: 32 atrophy-flagged versus 37 unflagged. After excluding ventilator cases: 24 versus 37. Flagging uses the recorded Pathology Categories field. FDR was applied separately across eight comparisons in each set. Unflagged specimens are not confirmed normal controls; 35 had no recorded category. These analyses do not establish independent validation of the grouping labels or hold death circumstances constant.

**S4a. Hardy category by GTEx stratum (n=398).**

| **Hardy Scale** | **General diseases** | **Very healthy** | **Wasting diseases** |
| --- | --- | --- | --- |
| Fast death - natural causes | 0 | 93 | 4 |
| Fast death - violent | 0 | 14 | 1 |
| Intermediate death | 0 | 0 | 12 |
| Slow death | 0 | 0 | 44 |
| Ventilator case | 222 | 0 | 8 |

Hardy categories describe death circumstances, not postmortem interval. Their limited overlap across groups prevents reliable separation of group and agonal-state associations.

**S4b. General-disease minus healthy differences, adjusted for age and sex.**

| **Indicator** | **Difference in SD units (95% CI)** | **p (no FDR)** |
| --- | --- | --- |
| MyoPath Score | 0.238 (-0.003–0.479) | 0.0538 |
| NCI | -0.003 (-0.243–0.238) | 0.983 |
| Fibre CV | 0.171 (-0.068–0.409) | 0.162 |
| Shape regularity | 0.174 (-0.068–0.417) | 0.16 |
| Fat infiltration | 0.379 (0.137–0.621) | 0.0023 |
| Fibrosis | 0.174 (-0.070–0.419) | 0.164 |
| Nuclear/muscle ratio | 0.080 (-0.166–0.326) | 0.523 |
| Connective nuclear density | 0.054 (-0.192–0.300) | 0.668 |

General-disease n=222; healthy n=107. Linear models used standardised indicators. These groups differ in illness and Hardy category, so coefficients are not isolated effects of ventilation. The p-values are not adjusted for multiple comparisons; non-significance does not establish equivalence.

**S5a. Variance inflation factors.**

| **Indicator** | **GTEx training set** | **HuashanMuscle** |
| --- | --- | --- |
| Fibre CV | 1.84 | 2.72 |
| NCI | 1.81 | 2.34 |
| Fibrosis | 1.6 | 3.08 |
| Fat infiltration | 1.5 | 1.69 |
| Shape regularity | 1.28 | 1.9 |
| Nuclear/muscle ratio | 1.13 | 1.88 |
| Connective nuclear density | 1.11 | 1.24 |

**S5b. Stability of standardised model coefficients.**

| **Indicator** | **β (95% bootstrap interval)** | **Sign consistency** |
| --- | --- | --- |
| NCI | 0.575 (0.111–1.094) | 0.989 |
| Fibre CV | 0.534 (0.042–1.183) | 0.98 |
| Fibrosis | 0.385 (-0.075–0.901) | 0.952 |
| Shape regularity | 0.218 (-0.178–0.622) | 0.849 |
| Fat infiltration | 0.164 (-0.254–0.601) | 0.771 |
| Nuclear/muscle ratio | 0.136 (-0.288–0.493) | 0.756 |
| Connective nuclear density | 0.051 (-0.434–0.476) | 0.579 |

1,000 stratified refits in the GTEx training set using L2 regularisation (C=1.0). Sign consistency is the fraction of refits retaining the point-estimate sign. Intervals describe sampling uncertainty conditional on the assigned group labels.

**S6. Calibration of the fixed GTEx-trained model.**

| **Setting** | **n** | **Case proportion** | **Mean predicted** | **Intercept** | **Slope** | **Brier score** |
| --- | --- | --- | --- | --- | --- | --- |
| GTEx training | 176 | 0.392 | 0.392 | 0.013 | 1.027 | 0.1745 |
| HuashanMuscle transfer | 79 | 0.785 | 0.487 | 2.716 | 1.606 | 0.2299 |

The intercept and slope are jointly estimated from logit(P(case)) = intercept + slope × original model logit. Perfect calibration corresponds to intercept 0 and slope 1. HuashanMuscle case proportion reflects the selected biopsy sample, not population prevalence. The transferred model was not retrained or recalibrated before its scores, AUCs or Brier scores were calculated.
